# Clinical feasibility of motor hotspot localization based on electroencephalography using convolutional neural networks in stroke

**DOI:** 10.1186/s12984-025-01736-3

**Published:** 2025-09-26

**Authors:** Ga-Young Choi, Jeong-Kweon Seo, Kyoung Tae Kim, Won Kee Chang, Sung Whan Yoon, Nam-Jong Paik, Won-Seok Kim, Han-Jeong Hwang

**Affiliations:** 1 Research Institute of Data Science and AI, Hallym University, Chuncheon, Republic of Korea; 2 Department of AI Convergence, Hallym University, Chuncheon, Republic of Korea; 3 Graduate School Innovation Team, Korea University, Seoul, Republic of Korea; 4 Department of Rehabilitation Medicine, Keimyung University School of Medicine, Keimyung University Dongsan Hospital, Daegu, Republic of Korea; 5 Department of Rehabilitation Medicine, Seoul National University College of Medicine, Seoul National University Bundang Hospital, Seongnam-si, 13620 Republic of Korea; 6 Graduate School of Artificial Intelligence, Ulsan National Institute of Science and Technology (UNIST), Ulsan, Republic of Korea; 7 Departmet of Electronics and Information Engineering, Korea University, Sejong, 30019 Republic of Korea; 8 Interdisciplinary Graduate Program for Artificial Intelligence Smart Convergence Technology, Korea University, Seoul, Republic of Korea

**Keywords:** Stroke, Deep learning, Motor hotspot, Electroencephalography, Neuromodulation, Neurorehabilitation

## Abstract

**Background:**

Although transcranial magnetic stimulation (TMS) is the optimal tool for identifying individual motor hotspots–specific regions of the brain that are essential for controlling voluntary muscle movements–it involves a cumbersome procedure that requires patients to visit the hospital regularly and relies on expert judgment. To address this, we propose an advanced electroencephalography (EEG)-based motor hotspot identification algorithm using a deep-learning and assess its clinical feasibility and benefits by applying it to EEGs for stroke patients, considering the noticeable variations in EEG patterns between stroke patients and healthy controls.

**Methods:**

Motor hotspot locations were estimated using a two-dimensional convolutional neural network (CNN) model. We utilized various types of input data, depending on the five processing levels, the five types of input data, depending on the processing levels, to assess the signal processing capability of our proposed deep-learning model using EEGs of thirty healthy subjects measured during a simple hand movement task. Furthermore, we applied our proposed deep-learning algorithm to the hand-movement-related EEGs of twenty-nine stroke patients.

**Results:**

The mean error distance between the motor hotspot locations identified by TMS and our approach for healthy subjects was 0.35 ± 0.04 mm when utilizing power spectral density (PSD) features. The mean error distance was 2.27 ± 0.27 mm for healthy subjects and 1.64 ± 0.14 mm for stroke patients, when using raw data without any feature engineering. Our proposed motor hotspot identification algorithm showed robustness concerning the number of electrodes; the mean error distance was 2.34 ± 0.19 mm when using only 9 channels around the motor area for healthy subjects, and 1.77 ± 0.15 mm using only 5 channels around the motor area for stroke patients.

**Conclusion:**

We demonstrate that our EEG-based deep-learning approach can effectively identify individual motor hotspots, and the clinical feasibility of our algorithm by successfully applying the proposed approach to stroke patients. It can be used as an alternative to TMS for identifying motor hotspots, potentially enhancing the effectiveness of rehabilitation strategies.

**Supplementary Information:**

The online version contains supplementary material available at 10.1186/s12984-025-01736-3.

## Introduction

According to a World Health Organization (WHO) report, stroke-related mortality reached 6.6 million cases in 2019, ranking as the third leading cause of death globally [1]. Stroke primarily leads to motor impairments that not only hinder daily activities but also significantly restrict social engagements, impacting the quality of life for both patients and caregivers, and potentially leading to chronic health issues. Therefore, understanding these challenges and developing effective rehabilitation programs are crucial. One of the efficient neurorehabilitation methods is transcranial electrical stimulation (tES), which is a non-invasive brain stimulation technique to modulate neuronal excitability, including transcranial direct current stimulation (tDCS) and transcranial alternating current stimulation (tACS) [1–5]. Evidence from numerous studies supports the crucial role of tES in improving motor function after stroke [6–9], particularly in enhancing hand movement skills [10–14].

Standard tES typically comprises a single anode and cathode electrode. To enhance hand motor function in stroke patients, the anode electrode is positioned on the primary motor cortex (M1) in the contralateral hemisphere of the paralyzed hand, whereas the cathode electrode is positioned either on the ipsilateral M1 site or the supraorbital region of the same hand. For example, in order to enhance right-hand motor function, the anode electrode is placed on the M1 site in the left hemisphere, with the cathode electrode on either the M1 or supraorbital region in the right hemisphere [15–18].

Identifying the precise M1 region for hand movement involves two methods. The first approach utilizes the international 10–20 system to estimate the location of the hand knob, which is the anatomical region governing hand movements [16, 19, 20]. The hand knobs are located in the central lobes of both hemispheres, referred to as C3 and C4 in the international 10–20 system, respectively. This approach is quick and convenient, but provides a rough estimation to identify brain regions responsible for hand movements. On the other hand, one study demonstrated the importance of precisely stimulating a specific motor area functionally governing hand movements, termed the motor hotspot [21]. Stimulation of the motor hotspot can enhance corticomotor excitability and motor skills more effectively than stimulation of the hand knobs, and thus, many recent studies have adopted this approach for motor rehabilitation [15, 21–25]. The motor hotspot, functional regions responsible for governing hand movements, is typically located anteriorly and laterally relative to the hand knob, and its location varies among individuals [26, 27].

### Related works

There are various methods for identifying individual motor hotspots. The conventional approach is to use transcranial magnetic stimulation (TMS), where the individual motor hotspot is identified when TMS elicits maximum motor evoked potential (MEP) in at least half of the attempts [28–31]. Transcranial magnetic stimulation (TMS) has been widely adopted not only as a diagnostic and mapping tool for localizing motor hotspots but also as a clinically validated neuromodulation technique to promote motor recovery following a stroke. Recent studies have demonstrated that stimulating individualized motor hotspots—typically identified through TMS-induced motor evoked potentials (MEPs)—can lead to more targeted and effective modulation of neural circuits [21, 32]. These findings underscore the increasing clinical significance of precise motor hotspot targeting across various neuromodulation modalities. However, this procedure is iterated multiple times to pinpoint the precise location of the motor hotspot, which is time-consuming and requires manual intervention by an expert. To address these challenges, recent studies have focused on improving practicality by implementing an automated motor hotspot identification technique that leverages a TMS system with a robot arm [33, 34]. For example, Meincke et al. proposed a closed-loop-based automated motor hotspot identification algorithm that continuously adjusts stimulation intensity based on real-time feedback using resting motor thresholds [34]. Despite these advancements, the TMS-based approach still requires expert knowledge and specialized equipment, including TMS, electromyography (EMG), and a robot arm, among others.

As an alternative to TMS-based approaches, functional magnetic resonance imaging (fMRI) can be utilized for identifying motor hotspots. fMRI measures blood oxygenation level-dependent (BOLD) signals while participants perform simple hand movement tasks. The brain regions showing the most significant activation of the BOLD signal are then defined as motor hotspots [35, 36]. In addition to fMRI, structural MRI (sMRI) has been occasionally used for the precise identification of motor hotspots. For example, Matilainen et al. constructed a customized computational head model based on individual sMRI data and applied the finite element method to model the electric field induced by TMS [37]. By inputting the MNI standard coordinates of the first dorsal interosseous (FDI) muscle into the head model, they attempted to estimate the individual motor hotspot location. This combination of fMRI and sMRI allows for the precise identification of individual motor hotspots by leveraging both functional and anatomical information. Despite the advantages of MRI-based techniques, MRI examinations have several limitations, including high costs, lengthy examination times, discomfort due to noise and enclosed spaces, and issues for individuals with metallic implants (e.g., pacemakers, cerebral aneurysm clips, and cochlear implants).

In our previous study, we introduced a machine learning-based algorithm that utilized electroencephalography (EEG) to automatically identify individual motor hotspots, thus eliminating the need for TMS and EMG. Integrating commercially available portable tES-EEG devices (e.g., Starstim tES-EEG systems by Neuroelectrics and M×N-5/M×N-9 HD-tES by Soterix Medical) with the developed algorithm could enhance the accessibility of tES rehabilitation [38–40]. This advancement might facilitate home-based neurorehabilitation for patients with limited mobility by using EEG to automatically pinpoint individual motor hotspots, potentially improving the efficiency of motor rehabilitation by substituting the costly TMS and MRI equipment with more affordable EEG technology. This approach not only simplifies the neurorehabilitation process but also provides economic advantages. However, our previous study validated the feasibility of the EEG-based motor hotspot identification approach only in healthy individuals. Further research is required to verify the effectiveness of our proposed motor-hotspot identification method in stroke patients because lesion location and severity can alter EEG patterns [41].

### Significance of study

In this study, we propose a more robust and convenient algorithm based on deep learning for identifying EEG-based motor hotspots adapted to the individual characteristics of stroke patients. We also aimed to simplify the process of domain-knowledge-based feature engineering, involving signal processing and feature extraction, by utilizing an algorithm based on a convolutional neural network (CNN). To do this, we initially assessed the motor hotspot identification performance of the CNN model by testing its signal processing capability on EEG data from healthy individuals; we evaluated the performance of the CNN model across various levels of handcrafted signal processing and then examined how performance varied by reducing the number of channels and trials to assess the robustness of the proposed model. Finally, given the distinguishable differences in EEG patterns between stroke patients and healthy controls [42–45], we validated the clinical feasibility by applying our novel motor-hotspot identification method to EEG data acquired from stroke patients. Our contributions in this paper are briefly summarized:

i) enhancing the performance of the EEG-based motor hotspot identification method.
ii) studying whether the level of preprocessing affects CNN performance.
iii) investigating practical usability in terms of the number of channels and trials.
iv) verifying the clinical feasibility of the proposed EEG-based motor hotspot identification method with stroke patients.

## Methods

The EEG dataset used in this study were previously reported in a preprint version of this work [40]. In the present manuscript, we have refined the analyses and provided a more comprehensive interpretation to extend the contribution beyond the preliminary version.

### Subjects

Thirty healthy subjects (10 females and 20 males; 25 ± 1.39 years; all right-handed) were recruited for this study. Based on the central limit theorem, a sample size of approximately thirty is often considered sufficient to approximate normality in the sampling distribution of the mean [46, 47]. Additionally, recent review studies have indicated that an average of 20 subjects is commonly enrolled to verify experimental hypotheses [48]. The participants had no history of psychiatric or neurological disorders that could affect the research outcomes. Before the experiment, participants were informed about the experimental procedure and required to sign an informed consent form. Adequate reimbursement was provided for their participation in the experiment. The study protocol was approved by the Institutional Review Board (IRB) of Kumoh National Institute of Technology (No. 6250). The study was conducted in accordance with the Code of Ethics of the World Medical Association (Declaration of Helsinki).

### Traditional motor hotspot identification by TMS

Before conducting EEG measurements, we identified the motor hotspots in both hands for each subject by analyzing the TMS-induced MEPs of the first dorsal interosseous (FDI) muscle. We used Ag-AgCl disposable electrodes (actiChamp, Brain Products GmbH, Gilching, Germany) to measure the MEPs. We used single-pulse TMS on the contralateral motor cortex (REMED, Daejeon, Korea) to find the motor hotspot. To identify the motor hotspot, we conducted a systematic search along an anterior-posterior region, beginning approximately two knuckles distal to the Cz electrode. The motor hotspot was defined as the area where the maximum MEP of at least 50 µV was elicited in more than 5 out of 10 consecutive stimuli, using the individual’s minimum stimulation intensity [28–31]. If multiple sites met this criterion, we selected the one that produced the highest MEP amplitude at the lowest intensity.

To record the locations of motor hotspots, we used a digitizer (Polhemus Inc., Colchester, Vermont, USA) to mark the 3D coordinates based on the vertex (Cz) in the international 10–20 system. The coordinates served as the ground truth for comparison with the estimates obtained through our EEG-based motor hotspot identification approach.

### Experimental protocol

**Fig. 1:**
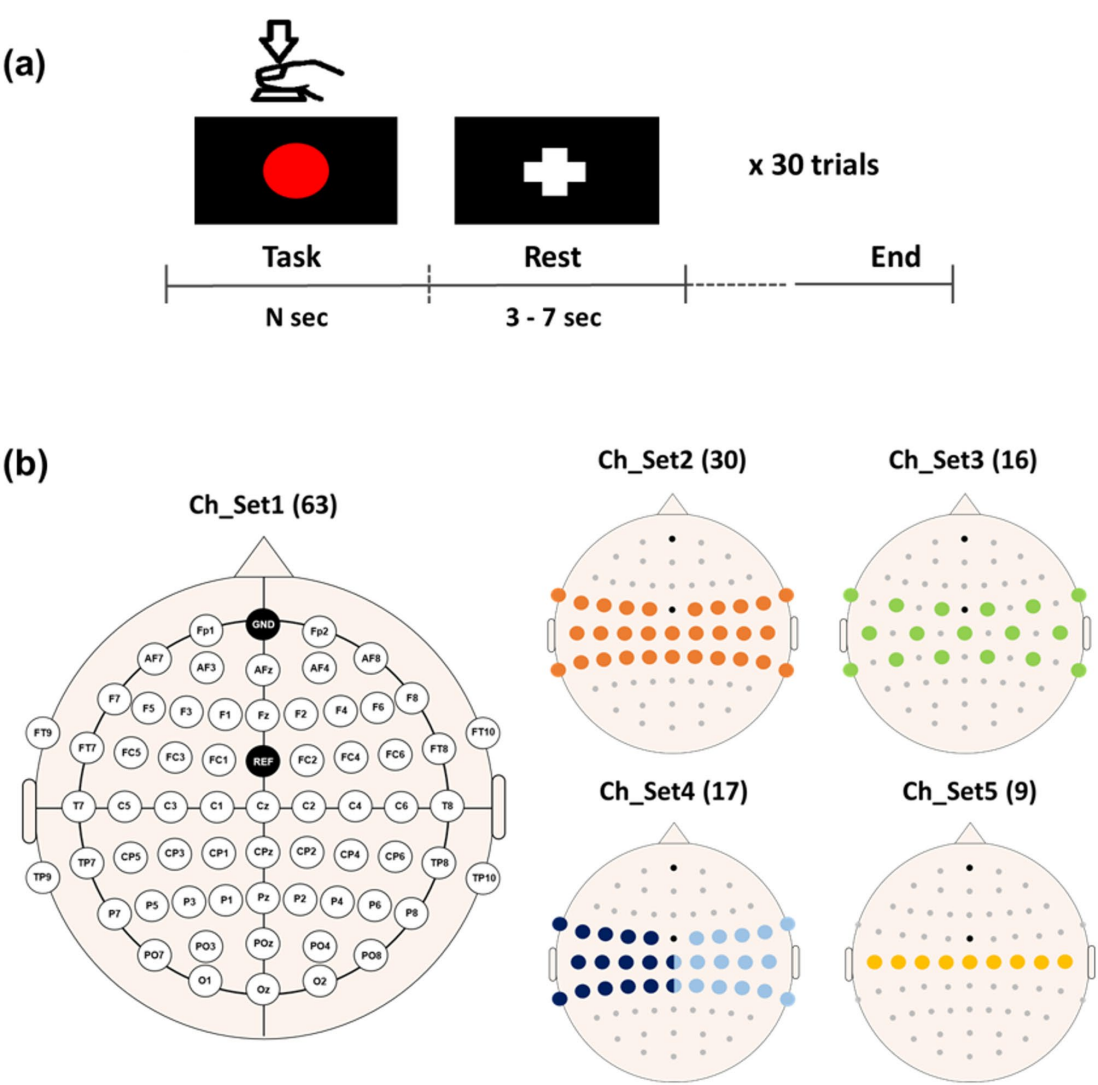
(**a**) Experimental paradigm. Each healthy subject pressed a space bar whenever the red circle appeared on the center of a monitor. The red circle remained visible until the subject pressed the space bar. At the end of the task period, a fixation (‘+’) mark was displayed to indicate a rest period. (**b**) Electrode location for EEG data acquisition in healthy subjects. Five different channel sets were used for data analysis to investigate the impact of the number of channels on the performance of the proposed motor hotspot identification algorithm. Channels in different colors in Ch_Set4 represent the selected channels in the contralateral motor cortex for the data analysis of each hand

The subjects performed a simple finger-tapping task to collect movement-related EEG data. They were instructed to press the spacebar with their index fingers whenever a red circle appeared in the center of a monitor (Fig. 1(a)). Each trial, involving a hand movement task followed by a relaxation period of 3 to 7 s, was performed 30 times for each hand, with sufficient breaks to prevent excessive fatigue.

### EEG recording

Sixty–three EEG electrodes were placed on the scalp according to the international 10–20 system to measure EEG data during a simple motor execution task. The ground and reference electrodes were attached to Fpz and FCz, respectively (Fig. 1(b)). EEG data were measured at a sampling rate of 1,000 Hz using a multi-channel EEG acquisition system (actiChamp, Brain Products GmbH, Gilching, Germany). EEG measurements were separately conducted for the index finger of the left and right hands, respectively. They were instructed to stay relaxed and to avoid any unnecessary movements during the experiment to reduce physiological artifacts. Prior to this experiment, the right hand was only used for first two subjects in preliminary experiments to verify the experimental paradigm. In addition, we excluded the EEG data of one subject for both hands and the left-hand data of three other subjects due to significant EEG data contamination from physiological artifacts. Thus, 29 and 25 EEG datasets were used for the right and left hand, respectively, for data analysis.

### Data analysis

#### Pre-processing

EEG data preprocessing was performed using the EEGLAB toolbox in MATLAB 2017b (MathWorks, Natick, MA, USA). Raw EEG data were downsampled to 200 Hz to streamline calculations. We applied a common average re-reference (CAR), which emphasizes the relative potential between electrodes by calculating the average potential across all electrodes; we subtracted the potential value of each electrode from the average to minimize inter-electrode noise and improve the spatial resolution of the EEG signal [49]. Subsequently, we used a zero-phase 3rd-order Butterworth filter to bandpass the signal from 1 to 55 Hz, which operates bidirectionally to prevent phase distortion in the signal [50, 51]. The filtered data underwent independent component analysis (ICA) to eliminate physiological artifacts. Specifically, ICA was applied to decompose the signal into independent components and isolate non-neurological artifacts, such as those associated with eye blinks or cardiac activity [52, 53]. We utilized the “runica” function provided by the EEGLAB toolbox, implemented in MATLAB [54]. Contaminated IC components were identified through visual inspection and were then eliminated based on their artifact characteristics. EEG patterns were cross-checked before, during, and after ICA application following the EEGLAB guidelines with a conservative rejection criterion [55]. Consequently, ICA was then applied to the filtered EEG data to remove physiological artifacts, where an average of 20.0 ± 6.66 components was removed across the subjects (median = 21). After preprocessing, the EEG data were then segmented between − 0.5 and 0.5 s based on the key press point for each trial. Power spectral densities (PSDs) of each trial were estimated in the gamma frequency bands (30–50 Hz) because gamma activity was identified as optimal features for identifying motor hotspots using EEG in our previous study [38]. Note that gamma activity is closely related to motor functions, along with alpha and beta activities [56–58]; a study demonstrated common gamma activity patterns in electrocorticography (ECoG) and EEG measurements over the sensorimotor cortex [59]. The PSD estimation was performed at a 1 Hz resolution using the fast Fourier transform (FFT).

The extracted PSDs and the 3D coordinates of motor hotspots identified by TMS-induced MEPs were used as input for training the CNN model. To assess the impact of different levels of signal processing on the performance of the proposed CNN model, we tested five different types of input values, each progressively refined from the previous stage:

- Input_1: time-series raw EEG data segmented from − 0.5 s to 0.5 s relative to the key press event.
- Input_2: CAR-processed time-series EEG data from Input_1.
- Input_3: bandpass-filtered time-series EEG data from Input_2.
- Input_4: ICA-filtered time-series EEG data obtained from Input_3.
- Input_5: PSD features extracted from Input_4.

The detailed information of the five different inputs is summarized in Table 1. Based on EEG features, the CNN model computed the three-dimensional coordinates of the motor hotspot.

**Table 1:** Five different types of input values for training the motor hotspot identification model, each progressively refined based on the outcomes of the previous stage

Name	Contents	Format of input
Input_1	Raw	channel × time
Input_2	Re-referencing (common average re-reference: CAR)	channel × time
Input_3	CAR + band-pass filter (BPF)	channel × time
Input_4	CAR + BPF + independent component analysis (ICA)	channel × time
Input_5	CAR + BPF + ICA + fast Fourier transform	channel × PSDs

#### Two dimensional-convolutional neural network model

**Fig. 2:**
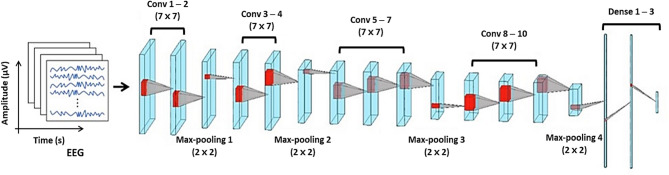
Schematic diagram of the proposed two dimensional-convolutional neural network (2D-CNN) for identifying EEG-based motor hotspot location. The triangle between two cubes visually represents the transitions between layers through convolution, pooling, and fully connected operations. This diagram illustrates the process by which the CNN model extracts features from input data, conducts dimensionality reduction, and generates predictions. Details of the layers are summarized in Table 2

Our proposed 2D-CNN model was implemented using the Keras library, an extension of TensorFlow based on the NVIDIA GeForce RTX 2080 SUPER graphical processing units (GPUs). As depicted in Fig. 2, the 2D-CNN model comprised 17 layers: 10 convolution layers, 4 pooling layers, and 3 fully connected layers [60, 61]. Convolutional layers use kernels to generate feature maps, whereas dimensionality is reduced in the 3rd, 6th, 10th, and 14th layers through max-pooling using two-window strides and zero-padding. All layers use the rectified linear unit (ReLU) activation function, except for the last fully connected layer, which uses a linear activation function to regress the locations of the motor hotspots. The detailed specifications of the proposed model for each layer, including kernel size and stride, are summarized in Table 2. The proposed model was trained using the adaptive moment (ADAM) optimization algorithm to minimize the mean square error (MSE) loss. Training parameters were set with a learning rate of 0.001, a batch size of 10, 1,000 epochs, and early stopping activated after 20 epochs without improvement in validation loss to avoid overfitting. The process of model parameter selection and optimization is detailed in the supplementary file (supplementary_results.doxs), and any parameters not mentioned were determined empirically.

**Table 2:** Details of the layer parameters in the proposed CNN model

**Layer**	**Layer Name**	**Kernel**	**Number of Filters**	**Stride**
0	Input	-	-	-
1	Conv1	7ⅹ7	16	1
2	Conv2	7ⅹ7	16	1
3	Max-pooling1	2ⅹ2	16	2
4	Conv3	7ⅹ7	32	1
5	Conv4	7ⅹ7	32	1
6	Max-pooling2	2ⅹ2	32	2
7	Conv5	7ⅹ7	64	1
8	Conv6	7ⅹ7	64	1
9	Conv7	7ⅹ7	64	1
10	Max-pooling3	2ⅹ2	64	2
11	Conv8	7ⅹ7	128	1
12	Conv9	7ⅹ7	128	1
13	Conv10	7ⅹ7	128	1
14	Max-pooling4	2ⅹ2	128	2
15	Flatten	-	-	-
16	Dense1	-	-	-
17	Dense2	-	-	-
18	Dense3	-	-	-

**Fig. 3:**
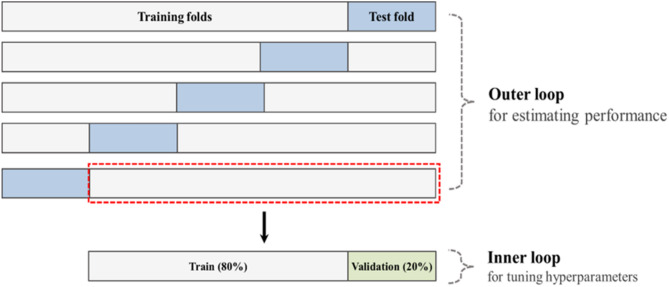
Scheme of the nested 5-fold cross-validation procedure

The 2D-CNN model was trained and tested using trial-wise-based nested cross-validation to build a user-adapted model that accounts for individual differences. Data from each subject (30 trials) were independently used for model training, as depicted in Fig. 3 [38]. In the nested cross-validation framework, a 5 × 5-fold cross-validation was implemented in the outer loop; data were randomly divided into 80% for training and 20% for testing. In the inner loop, the 80% training dataset from the outer loop was further partitioned into training and validation sets at a 4:1 ratio to determine optimal hyperparameters. The prediction performance was evaluated using the test dataset from the outer loop, and this procedure was repeated 25 times (5 × 5-fold) to enhance robustness and reliability. The performance of the final model was reported based on the averaged outputs across these iterations.

### Performance verification

The error distance was quantitatively estimated by calculating the Euclidean distance between the 3D coordinates of the motor hotspot identified by TMS-induced MEP and those determined by the EEG-based deep learning approach. Given that the human head shape is roughly spherical, using a spherical coordinate-based distance as a metric could be considered, but the Euclidean distance can provide a sufficiently accurate evaluation, as all motor hotspots were located in relatively flat motor areas that do not exhibit significant curvature [62–64]. Additionally, we investigated the impact of the number of channels and trials on the performance of motor hotspot identification. This analysis involved systematically reducing the number of channels and trials, with a focus on the central area associated with motor functions, as shown in Fig. 1(b).

### Verification of clinical benefits with stroke patients

#### Subjects

Stroke patients were enrolled from the rehabilitation department of a tertiary hospital. The study included twenty-nine patients with acute stroke (5 females and 24 males; 63 ± 12 years; all right-handed except for one subject) (Table 3 for detailed demographic data). This decision was made following the rationale mentioned in Sect. 2.1, aiming to match the recruitment numbers of both groups as closely as possible.

**Table 3:** Patient characteristics (*N* = 26); this table presents information on 26 out of the 29 participants recruited, with three individual being completely excluded from the analysis

Characteristics	*N* = 26
Age (years), mean (SD)	62 (12.4)
Sex, n (%)	
Male	22 (84.6)
Stroke Type, n (%)	
Ischemic	21 (80.8)
Hemorrhagic	3 (11.5)
Both	2 (7.7)
Hemiplegia side, n (%)	
Right	12 (46.2)
Lesion location, n (%)	
Cortical only	1 (3.8)
Subcortical only	11 (42.3)
Both	2 (7.7)
Brain stem	12 (46.2)
Time since stroke (days), median (IQR)	14 (12.0–19.0)
Upper extremity FMA, median (IQR)	53.5 (29.3–62.0)

SD, standard deviation; n, the number of patients; %, the percentage of the total population; FMA, Fugle Meyer Assessment; IQR, interquartile range

Inclusion criteria were as follows: (1) aged 18 to 85 with impaired upper limb function; (2) confirmed ischemic or hemorrhagic stroke via a neuroimaging technique, such as CT or MRI; (3) a score above 16 on the Korean Mini-Mental State Exam (K-MMSE); (4) ability to follow instructions for clinical assessment and EEG study. Exclusion criteria included: (1) traumatic brain injury or uncontrolled internal/surgical diseases; (2) other disorders, such as altered consciousness or psychiatric disorders; (3) pregnancy; (4) implanted pacemakers, cochlear implants, or a history of brain surgery. Participants were provided with information regarding the experimental procedure and signed informed consent forms to participate in the study. If the patient meets our criteria but is unable to provide consent due to a disability, a legal representative will provide consent on their behalf. Note that there were no patients in the study who were unable to provide consent. The study protocol was approved by the Seoul National University Bundang Hospital IRB (registration No.: B-1912-580-005) and adhered to the Code of Ethics of the World Medical Association (Declaration of Helsinki).

#### Experimental protocol

Disposable Ag-AgCl electrodes (Synergy EMG/EP system; Oxford Instruments Medical Ltd., Surrey, UK) were attached to the patients’ FDI muscle, and a single TMS pulse (MagPro X100; MagVenture, Farum, Denmark) was applied to identify the motor hotspot location for each hand, following the procedure outlined in **‘2.2. Traditional Motor Hotspot Identification by TMS’** prior to the experiment. If a unilateral MEP was only identified, the single hotspot location was recorded for the patient.

**Fig. 4:**
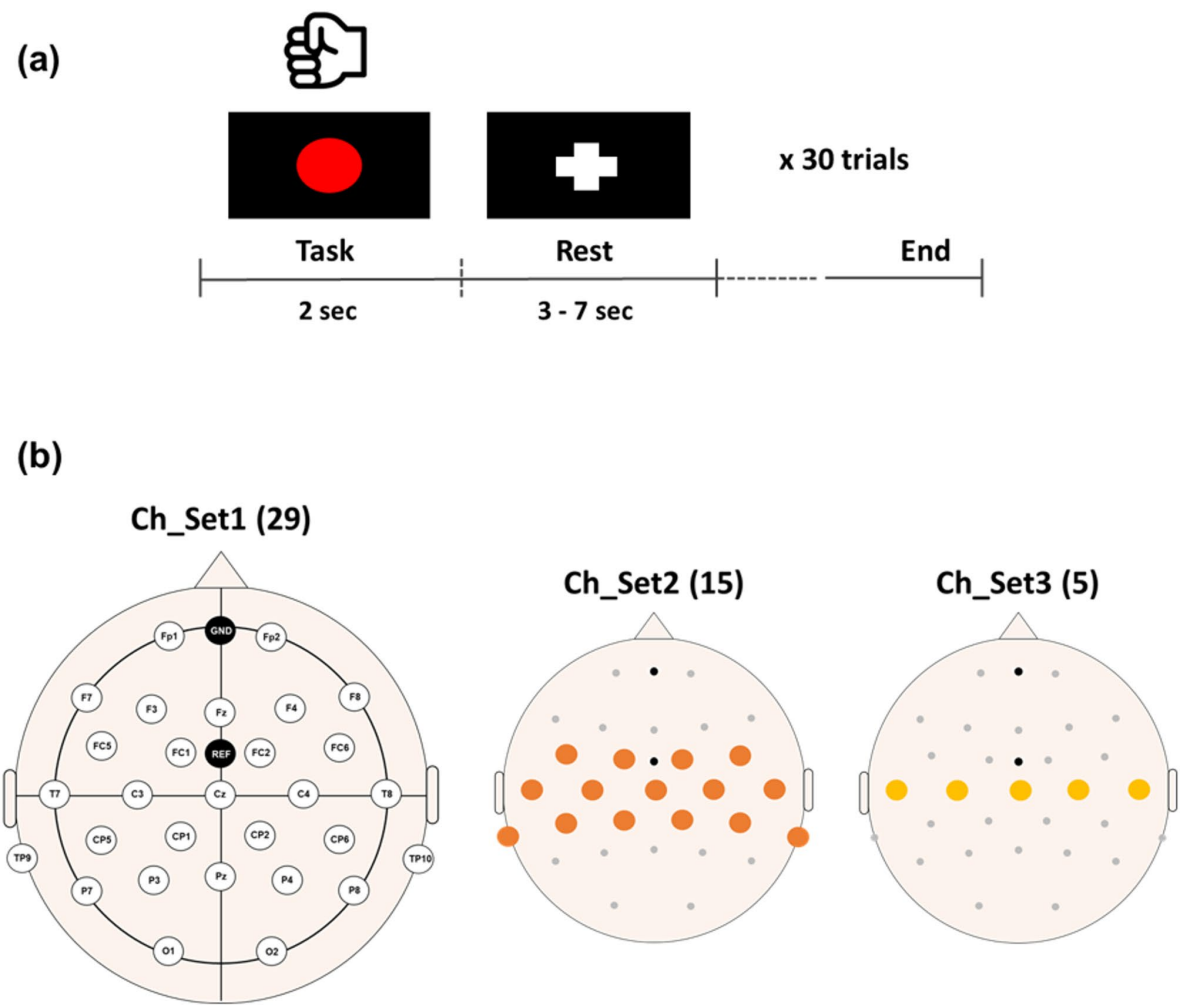
(**a**) Experimental paradigm for stroke patients. Each patient was instructed to grip their hand within 2 s upon the appearance of a red circle. At the end of the task period, a fixation (‘+’) mark was displayed to indicate a rest period. (**b**) Electrode location for EEG data acquisition in stroke patients. Three different channel sets are used for data analysis to investigate the impact of the number of channels on the identification performance of moto hotspot locations. Numbers in parentheses indicate the numbers of channels in each channel set

After identifying individual motor hotspots using TMS, EEG data were sampled at a rate of 500 Hz using a multi-channel EEG acquisition system (Liveamp, Brain Products GmbH, Gilching, Germany). The patients completed a simple hand movement task, detailed in **‘2.3. Experimental Protocol’**, alternating movement and rest across 30 trials for each hand. Due to limited motor ability in stroke patients, the finger-tapping task was replaced with a simple hand-grasp task, where the patients lightly grasped their hand whenever a red circle appeared in the center of the monitor (Fig. 4(a)). The twenty-nine EEG electrodes were attached to the scalp using the international 10–20 system, with ground and reference electrodes at Fpz and FCz, respectively (Fig. 4(b)). EEG data from three subjects for the affected hand and three subjects for the unaffected hand were excluded due to the absence of motor hotspot locations. Additionally, we excluded EEG data from three subjects for both hands due to recording errors and significant contamination from physiological artifacts. **Consequently**,** EEG data from 23 stroke patients were included for the affected hand condition**,** and another 23 for the unaffected hand condition. Among them**,** 20 subjects contributed data to both conditions**,** while the others were included in only one due to incomplete recordings related to the patients’ conditions.**

The recorded EEG data were analyzed as outlined in **‘2.5. Data Analysis’** and **‘2.6. Performance Verification’**. Exceptionally, the data were segmented between − 0.5 and 1 s based on the visual stimulation indicating the starting point of the task for each trial. This approach accommodated slight variations in task completion time across patients, though all completed within 1 s. In this analysis, only raw data (Input_1) were used to identify motor hotspots in stroke patients because the error distance for Input_1, while being the largest compared to other input data, was considered practically acceptable (less than 3 mm), as demonstrated in healthy individuals (see Fig. 6 for details in advance).

**Fig. 5:**
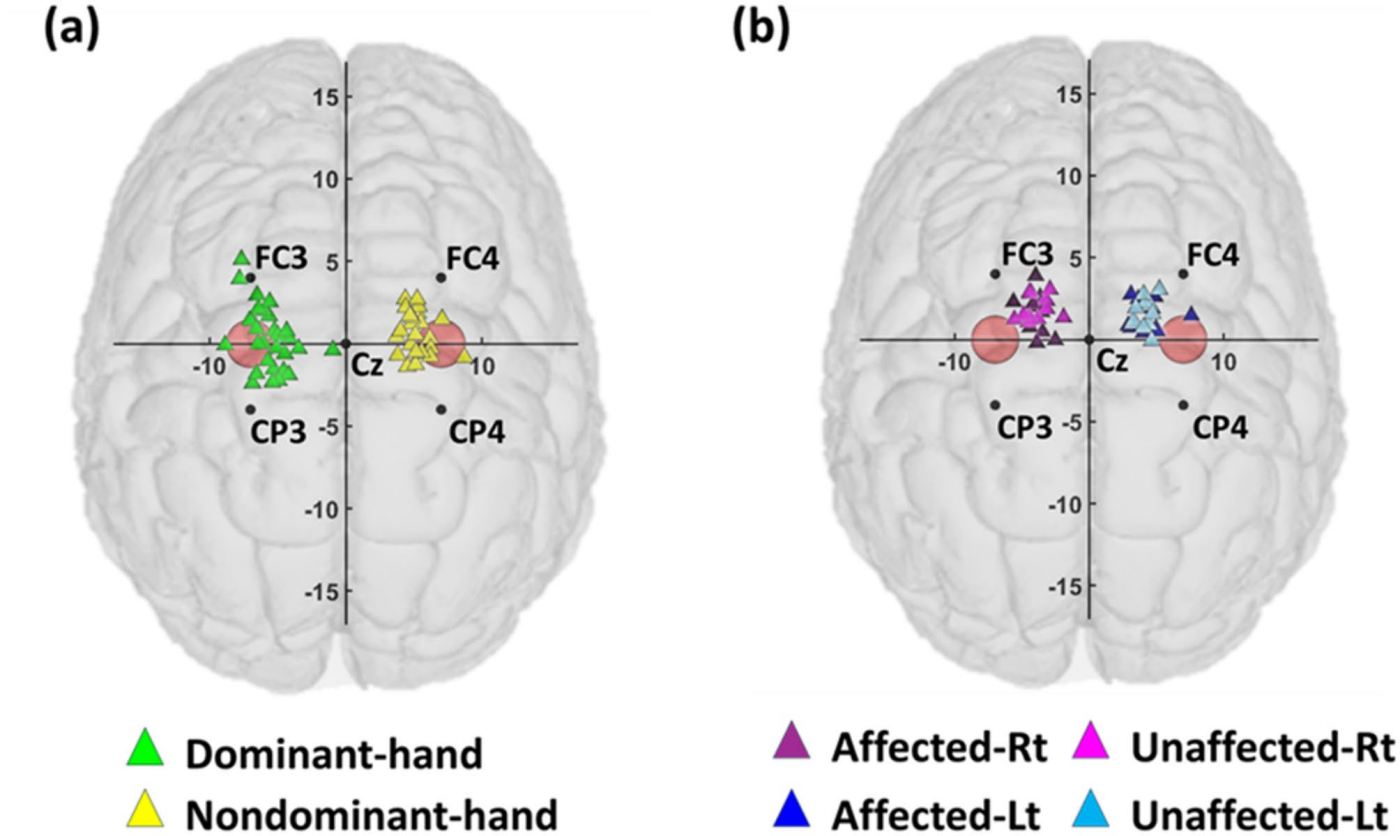
Motor hotspot locations identified by TMS-induced MEP for (**a**) healthy individual and (**b**) stroke patients. The red circles indicate the locations of C3 and C4 with a diameter of 10 mm, considering the minimum size of tES electrode, indicating that precise location identification is essential, as the location of the motor hotspot does not align with the C3/C4 and varies from individual to individual. The mean distances and standard errors between the red circle and the individual motor hotspot for each hemisphere are as follows: 25.22 ± 2.18 mm for the dominant hand of healthy individuals **(mean ± SE**,** N = 29)**, 23.25 ± 2.35 mm for the non-dominant hand of healthy individuals **(mean ± SE**,** N = 25)**, 37.47 ± 2.24 mm for the affected right hand (Affected-Rt) of stroke patients **(mean ± SE**,** N = 10)**, 32.29 ± 2.82 mm for the affected left hand (Affected-Lt) of stroke patients **(mean ± SE**,** N = 12)**, 36.27 ± 2.81 mm for the unaffected right hand (Unaffected-Rt) of stroke patients **(mean ± SE**,** N = 13)**, and 33.81 ± 1.74 mm for the unaffected left hand (Unaffected-Lt) of stroke patients **(mean ± SE, N = 11)**

## Results

Figure 5 presents the individual motor hotspot locations identified by TMS-induced MEP relative to C3 or C4, marked by 10 mm red circles. It is evident that the motor hotspots of most participants are not aligned with the location of C3 or C4 and are distributed outside the red circles, which is more commonly observed in stroke patients. The mean distance and standard error between the motor hotspots of healthy individuals and the C3 or C4 location in each hemisphere were found to be 24.31 ± 1.39 mm **(mean ± SE**,** N = 54)**, while it was 34.96 ± 1.25 mm for stroke patients **(mean ± SE**,** N = 46)**. The individual motor hotspots are located either more anteriorly or posteriorly than the hand knob (C3 or C4). These results indicate that individual motor hotspots can be located either anterior or posterior to the hand knob (C3 or C4), highlighting the importance of individualized hotspot identification [26, 65]. Moreover, we have verified distinct neurological patterns in the EEGs of stroke patients compared to the healthy group (see Supplementary Fig. 1) [42–45].

**Fig. 6:**
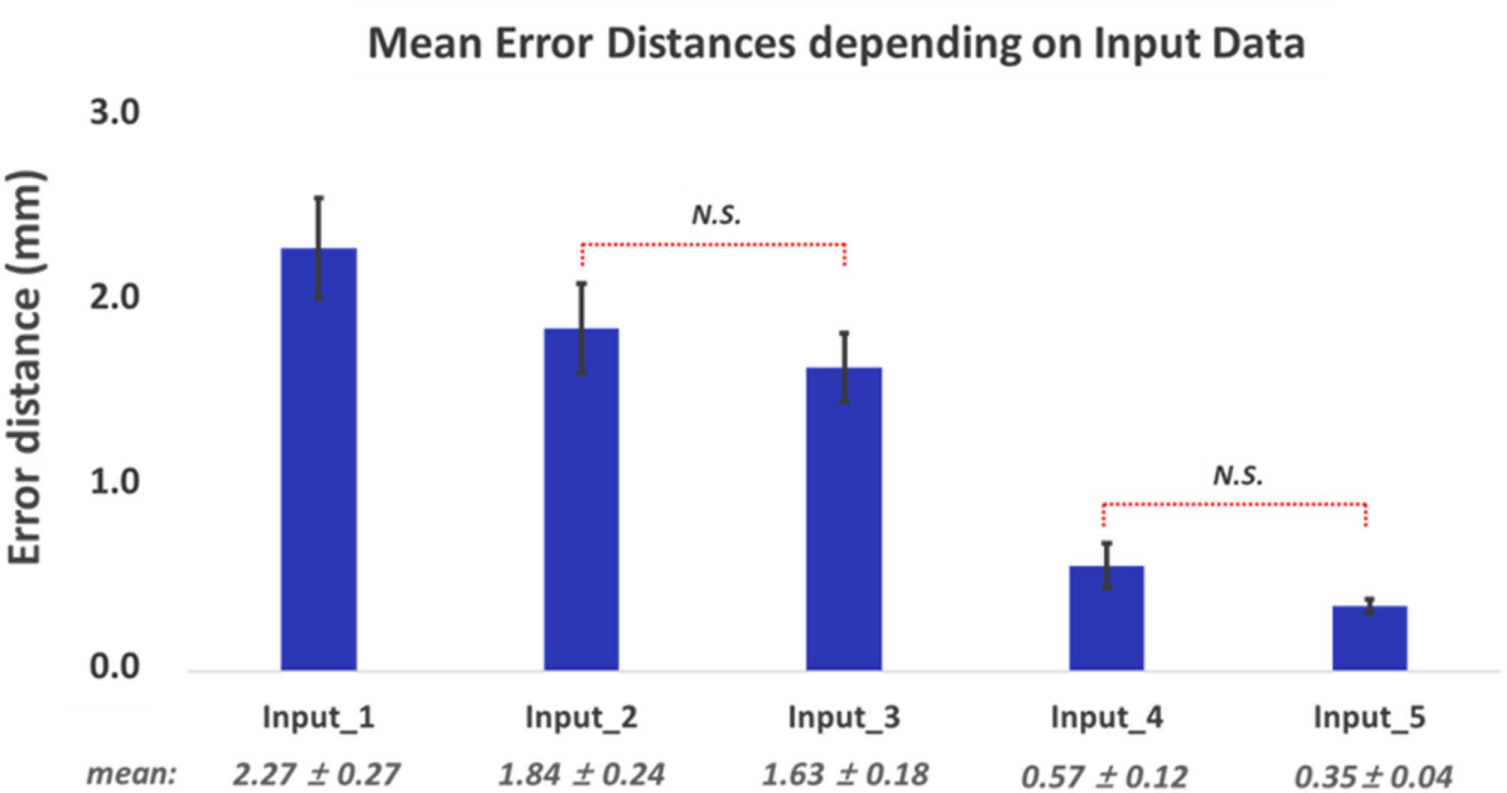
Mean error distances and standard errors (mean ± SE, N = 54) in identifying motor hotspot locations with respect to input data (Friedman test with Bonferroni corrected, p -value < 0.05: N.S. indicates no significance. All other pairs show significant differences

### Model validation in healthy subjects

Figure 6 shows the mean error distances of the estimated motor hotspot locations using the EEG-based deep learning approach with 63 channels from healthy controls with respect to the input types of the CNN method. Note that no significant difference was observed between the dominant and non-dominant hands for all conditions (Wilcoxon rank sum, *p*-value < 0.05), and thus only the mean results for both hands will be reported for all results of healthy subjects. The mean error distances consistently decreased as the raw data were processed with additional signal processing techniques (Friedman test, followed by Wilcoxon signed rank for post hoc with Bonferroni corrected, *p*-value < 0.05: N.S. means no significance, and the pairs without N.S. showed significant differences). Even though the input of raw data (Input_1) showed the largest mean error distance of 2.27 ± 0.27 mm, this error distance is still practically usable, considering that the diameter of commercial tES electrodes is typically at least 10 mm. Given that the error distance of the motor hotspot identified by Input_1 (raw data) was acceptable in practice, Input_1 was utilized for subsequent analyses.

**Fig. 7:**
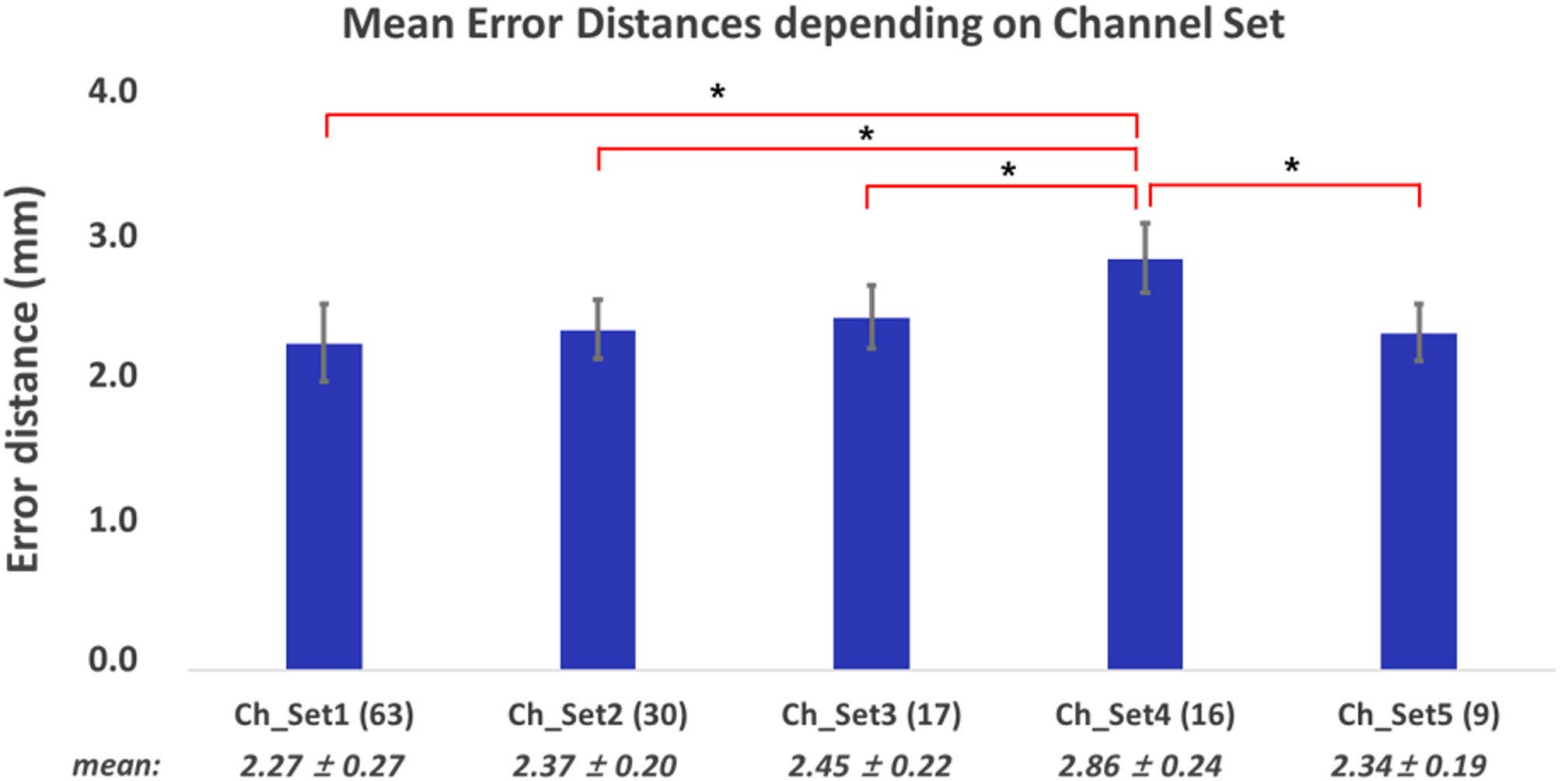
Mean error distances and standard errors **(mean ± SE, N = 54)** in identifying motor hotspot locations using raw-EEG with respect to the number of channels (Friedman test with Bonferroni corrected, p-value < 0.05)

Figure 7 presents the mean error distances of the estimated motor hotspot locations for both hands with respect to the number of channels using raw data (Input_1). The performance remained excellent, with a mean error distance of less than 3 mm under all conditions. Notably, the average error distance was 2.34 ± 0.19 mm, even when only using 9 channels around the motor area (Friedman test, followed by Wilcoxon signed rank for post hoc with Bonferroni corrected, *p*-value < 0.05: the red lines represent the pairs with significant differences. The pairs without lines showed no significant differences).

**Fig. 8:**
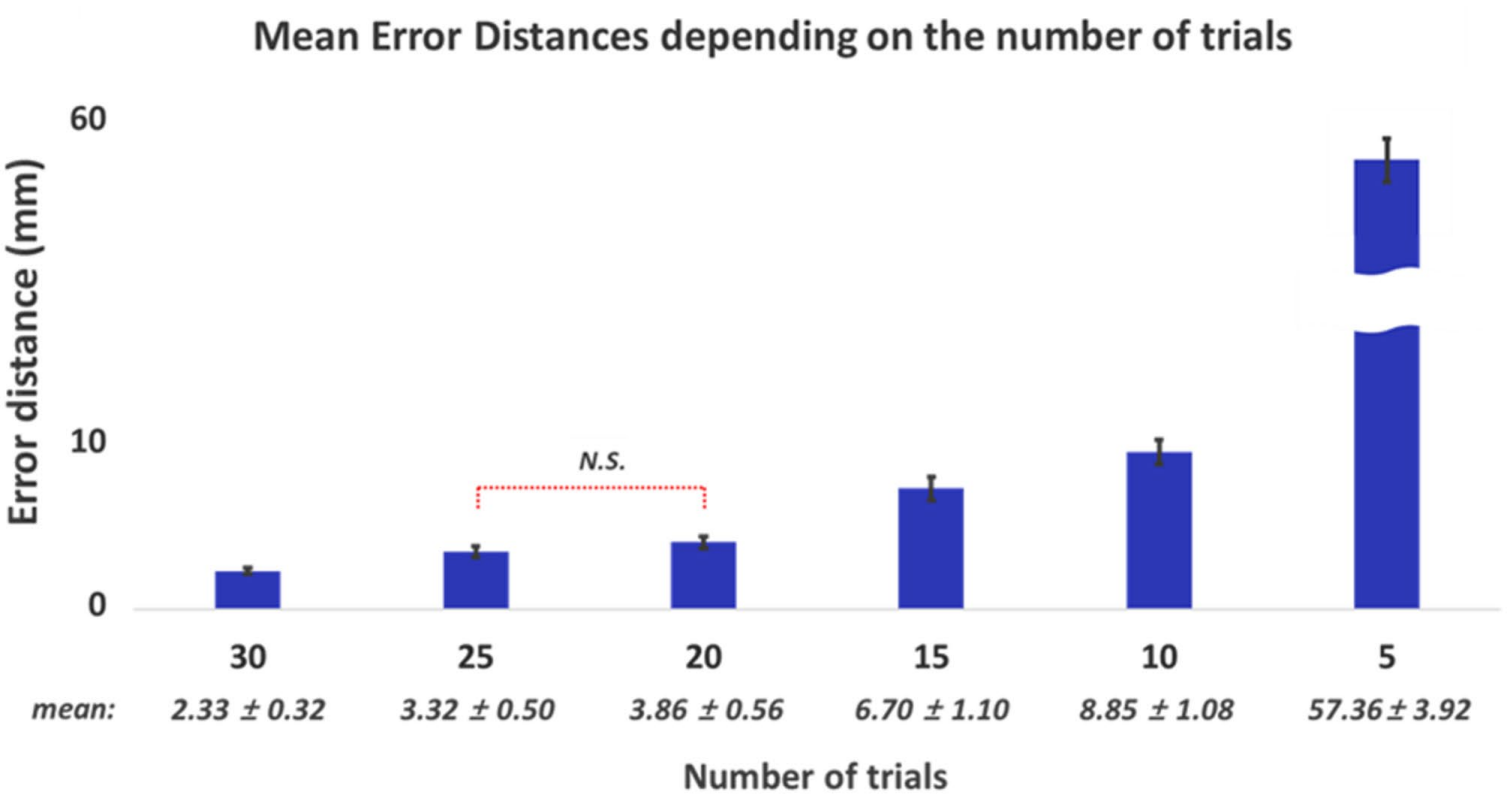
Mean error distances and standard errors **(mean ± SE**,** N = 54)** in identifying motor hotspot locations using 9 channels of raw EEG with respected to the number of trials (Friedman test with Bonferroni corrected, p-value < 0.05: N.S. indicates no significance. All other pairs show significant differences

Figure 8 presents the mean error distances of the estimated motor hotspot locations for both hands with respect to the number of trials using 9 channels of raw data. Although the mean error distances increased as the number of trials used for analysis was reduced, it is important to note that the mean error distance remained below 10 mm even when the number of trials was reduced to 10 (Friedman test, followed by Wilcoxon signed rank for post hoc with Bonferroni corrected, *p*-value < 0.05: N.S. means no significance, and the pairs without N.S. showed significant differences).

### Impact of input types on training and inference time

**Fig. 9:**
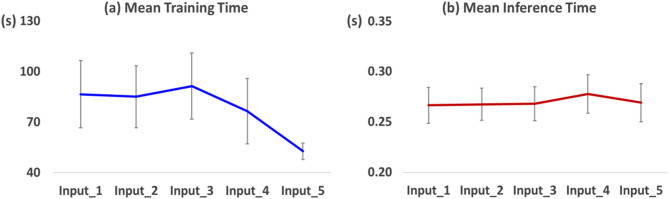
Mean (**a**) training time and (**b**) inference time with respect to input data, including standard deviation

Figure 9 shows the mean training and inference time with 63 channels from healthy subjects with respect to the input types of the CNN model. The mean training time decreased when the raw data were processed with additional signal processing techniques due to the removal of irrelevant information, such as physiological artifacts. However, no significant differences were observed across all conditions in both training and inference times (Friedman test, followed by Wilcoxon signed rank for post hoc with Bonferroni corrected, p-value > 0.05).

### Clinical validation in stroke patients

**Fig. 10:**
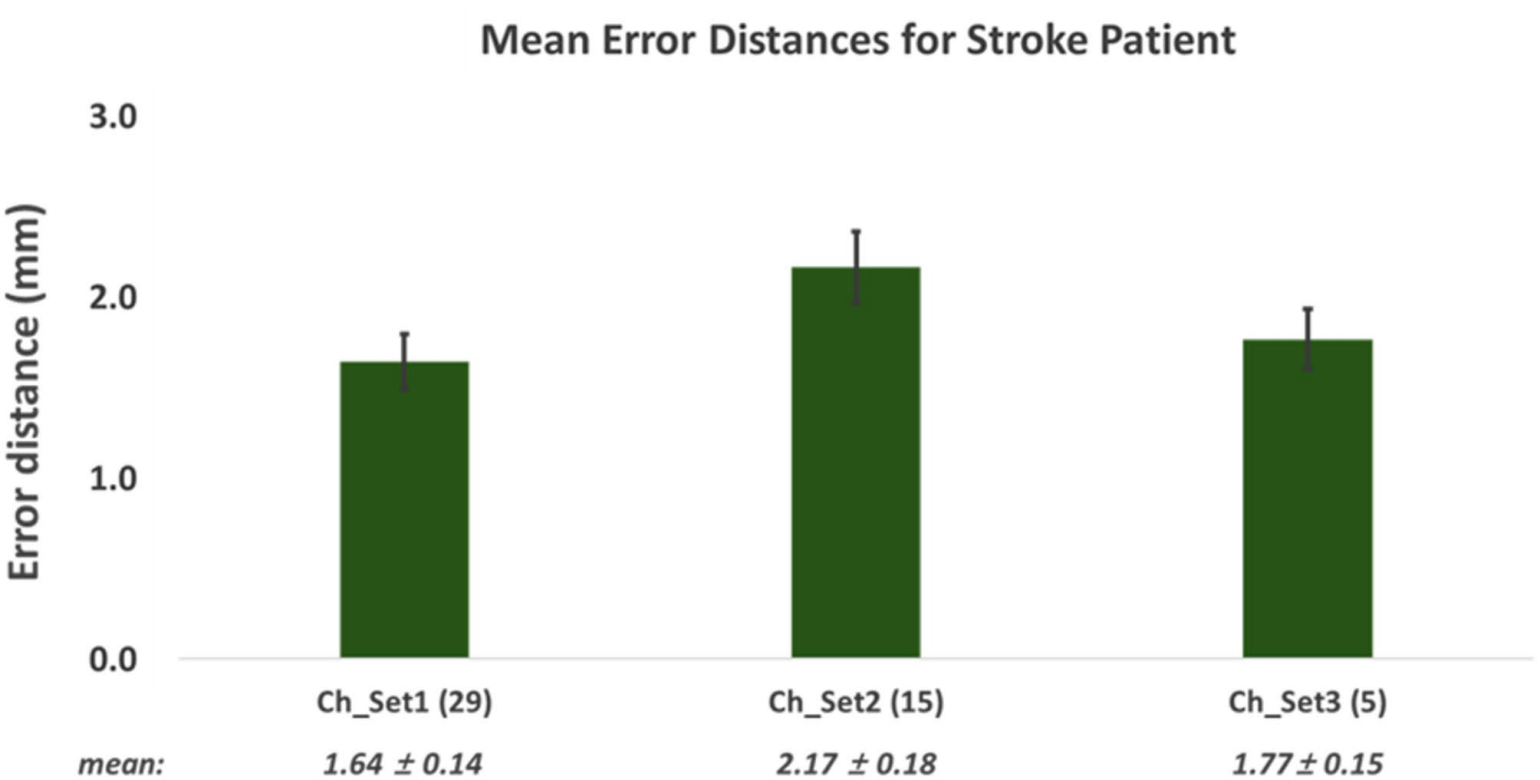
Mean error distances and standard errors **(mean ± SE**,** N = 46) **in identifying motor hotspot locations using raw EEG from the stroke patient with respect to the number of channels (Friedman test with Bonferroni corrected, p-value > 0.05)

Figure 10 presents the mean error distances of the estimated motor hotspot locations for both hands using raw data from stroke patients. Note that no significant difference was observed between the affected and unaffected hands for all conditions (Wilcoxon rank sum, p-value < 0.05), and thus only the mean results for both hands will be reported for all results of stroke patients. Despite the different neurological patterns observed in the EEGs of stroke patients compared to the healthy group (see Supplementary Fig. 1), all channel sets showed good mean error distances of approximately 2 mm. In particular, the error distance was only 1.77 ± 0.15 mm when using only 5 channels around the motor area, and there are no significant differences observed in relation to the number of channels used (Friedman test, followed by Wilcoxon rank sum for post hoc with Bonferroni corrected, p-value > 0.05).

**Fig. 11:**
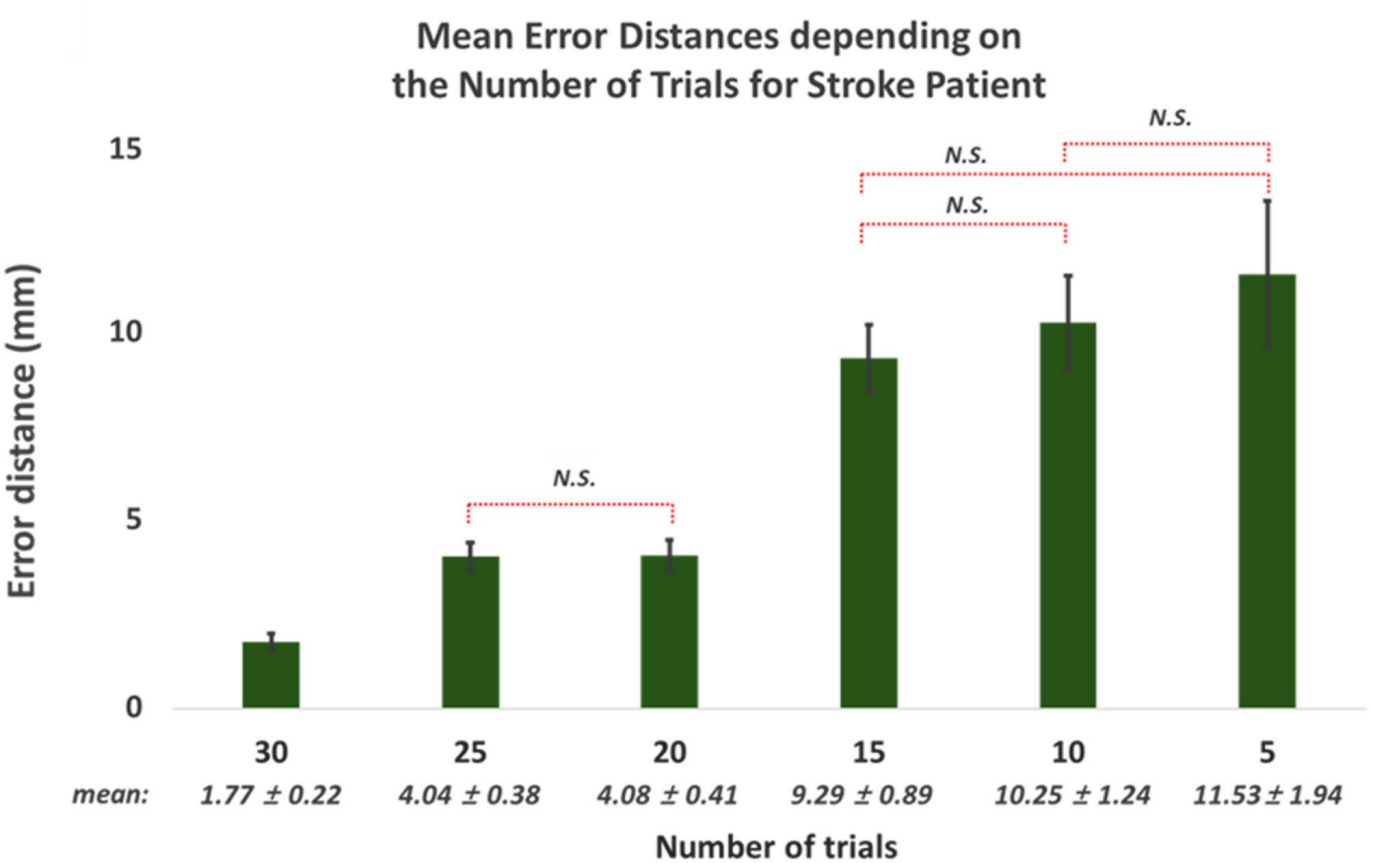
Mean error distances and standard errors **(mean ± SE**,** N = 46)** in identifying motor hotspot locations using 5 channels of raw EEG from the stroke patient with respected to the number of trials (Friedman test with Bonferroni corrected, p-value < 0.05: N.S. means no significance, and the pairs without N.S. means significant differences)

Figure 11 presents the mean error distances of the estimated motor hotspot locations for both hands with respect to the number of trials using 5 channels of raw data from stroke patients. Despite the increase in mean error distances with a decrease in the number of trials used for analysis, it is noteworthy that the mean error distance remained reliably below 10 mm even when the number of trials was limited to 15 (Friedman test, followed by Wilcoxon rank sum for post hoc with Bonferroni corrected, p-value < 0.05: N.S. means no significance, and the pairs without N.S. showed significant differences).

## Discussion

In this study, we proposed an advanced EEG-based motor hotspot identification algorithm using CNN structure, aiming at replacing the conventional motor hotspot identification method that uses TMS. The best mean error distance for healthy subjects was 0.35 ± 0.04 mm when utilizing PSD features (Input_5), which was superior to the best error distance of 2.18 ± 0.26 mm obtained using the artificial neural network (ANN)-based approach introduced in our previous study [38]. This demonstrates a substantial improvement in accuracy by approximately 82%. Most importantly, the best mean error distance for stroke patients was 1.64 ± 0.14 mm, which falls well within the spatial range typically associated with motor hotspot activation related to FDI (~ 18 mm of the hotspot center) [66], supporting the practical applicability of the proposed method in clinical settings.

### Advantages of our approach

We found that the locations of individual motor hotspots did not align with the hand knob (around C3 or C4) in both healthy and patient groups (Fig. 5). Considering that precise identification and stimulation of motor hotspots are more effective than using conventional criteria (C3, C4), this finding underscores the necessity for individual motor hotspot identification [21, 26]. Several previous studies have explored alternative methods to TMS for the identification of individual motor hotspots. Weiss et al. [35] attempted to identify motor hotspots based on fMRI data measured on healthy individuals, reporting a mean error distance of approximately 6.2 mm in comparison to the hotspot locations identified by TMS. Matilainen et al. used sMRI for motor hotspot identification and reported a mean error distance of 13 mm [37]. In contrast, our study achieved a mean error distance of 2.27 mm, even when using raw EEG data without any signal processing, demonstrating the effectiveness of our proposed method as an alternative to TMS for identifying individual motor hotspots. Moreover, our proposed EEG-based method is more cost effective and advantageous in terms of integration with tES compared to other modalities, such as TMS, sMRI, and fMRI.

In terms of clinical applicability, our proposed method can be easily implemented in home-based rehabilitation using existing commercial tES-EEG devices. These devices are designed for remote supervision and ease of use by non-experts, including patients and caregivers. With a short training period—typically just a few sessions—most users can operate the system correctly and safely. Importantly, our approach requires only an initial calibration; re-calibration is necessary only if the device is significantly repositioned, which significantly improves the feasibility of home use without frequent expert intervention. To improve real-world deployment, future work should consider conducting usability testing with patients and caregivers in home settings. In this respect, our method for identifying motor hotspots is thus well-suited to advance home-based motor rehabilitation using tES techniques, enhancing both accessibility and usability for individuals with mobility limitations and reducing the need for frequent clinical visits. As a result, patient compliance and engagement in neurorehabilitation are expected to increase, thereby improving functional outcomes. In addition, by replacing costly TMS systems with more affordable EEG-based solutions, our method may help lower the economic burden of neurorehabilitation and make these services more broadly available.

### Ablation study of the proposed model

We evaluated the signal processing capability of our proposed model based on the motor hotspot identification performance by varying the handcrafted signal processing level of the input data. Although there were statistical differences between different input types, the predicted error distance for all inputs remained within practical limits (< 10 mm). Moreover, the FDI-MEP can be evoked at a maximum distance of about 18 mm from the center of the motor hotspot [66]. These results demonstrate the effective signal processing and feature extraction capability of the proposed algorithm, indicating that practitioners without professional knowledge about EEG signal processing can utilize our method to accurately find motor hotspots.

In addition, we investigated the impact of the number of channels on the performance of motor-hotspot identification. The results indicated a slight quantitative difference in performance relative to the number of channels; however, the performance of motor-hotspot identification remained robust. In particular, using only nine channels around the motor area resulted in a mean error distance of 2.34 ± 0.19 mm. This contrasts with the result of our previous study using an ANN model, where mean error distances increased linearly as the number of channels decreased (2.27 ± 0.27 mm using 63 channels ◊ 13.2 ± 0.15 mm using 9 channels around the motor area). The practicality of the proposed algorithm was significantly enhanced, particularly regarding the time required for EEG electrode attachment, as a practically usable mean error distance was still obtained using only nine channels (< 10 mm).

Additionally, the proposed algorithm showed robust performance in terms of the number of trials; an acceptable mean error distance was maintained even when the number of trials was reduced by a third (2.34 ± 0.19 mm using 30 trials ◊ 8.85 ± 1.08 mm using 10 trials). However, the mean error distance linearly increased as the number of trials was reduced, reaching 57.36 ± 3.92 mm when using only five trials. To effectively utilize our proposed algorithm in clinical settings, using a minimal number of trials is advantageous for training the model since they are obtained from patients with motor impairments. Therefore, enhancing the robustness of our proposed model in terms of the number of trials is essential in future studies. Advanced data augmentation techniques, such as variational autoencoders, generative adversarial networks, and diffusion models, could be employed to address this issue by generating synthetic, yet realistic data without necessitating extensive data collection from patients.

As noted, our ablation study primarily focused on input representation, including variations in handcrafted features, the number of channels, and trials, as we were concerned with the practical application of the proposed model. The CNN model used in this study has been frequently applied in image processing [60]– [61], so we did not conduct an ablation study based on modifications to the model architecture. However, to further improve motor hotspot identification performance, fine-tuning the model or introducing a novel deep learning architecture would be beneficial, and in such cases, an ablation study on model architecture would be necessary.

### Model efficiency

We also investigated the mean training and inference time of our proposed motor hotspot identification model across different input types. Even when using the raw data obtained using all 63 EEG electrodes (Input_1), the mean training and inference time for our proposed model were approximately 86.5 s and 0.27 s, respectively. Notably, the training time decreased when the raw data were processed with additional signal processing techniques due to the removal of irrelevant information, such as physiological artifacts (Input_1: 86.5 s ± 20.0, Input_2: 85.0 s ± 18.4, Input_3: 91.3 s ± 19.5, Input_4: 76.4 s ± 19.4, and Input_5: 52.6 s ± 4.9). Importantly, the performance of motor hotspot identification was well retained despite a substantial reduction in the number of electrodes and trials. This indicates that the training time can be further reduced without a significant drop in performance, thereby making our proposed motor hotpot identification model practical for use with a delay of less than 1 min after EEG measurement.

### Clinical validation in stroke patients

On the other hand, the EEG patterns of patients with motor impairments may differ from those of healthy individuals [42–45], as confirmed in our data (see Supplementary Fig. 1). It is important to validate the proposed motor-hotspot identification method with patients to demonstrate its clinical feasibility, as variability in EEGs may lead to suboptimal locations of motor hotspots. Remarkably, the stroke group exhibited comparable motor-hotspot identification performance to that of healthy individuals. Furthermore, the acceptable mean error distance was preserved even with a reduction in the number of trials (1.77 ± 0.22 mm for 30 trials ◊ 9.29 ± 0.89 mm for 15 trials). These results demonstrate that our proposed EEG-based motor-hotspot identification method can be successfully transferred from healthy subjects to patient groups.

### Challenges and future directions

As shown in this study, CNN-based architectures have demonstrated significant potential as a foundation for EEG analysis; however, there remains room for improvement in model performance and adaptability. Consequently, future research will focus on integrating alternative deep learning architectures that can more accurately capture dynamic changes in long-term temporal information or effectively manage non-stationary signals. For instance, combining CNNs with RNNs or LSTMs may facilitate more precise modeling of the temporal evolution of brain states, while transformer-based models, which learn global dependencies, have the potential to enhance robustness against session-to-session variability. Such hybrid approaches could improve decoding accuracy and reduce the need for repeated calibration, thereby increasing the practicality of the system for long-term, self-directed neurorehabilitation applications. In addition, a multimodal approach may be considered to further enhance performance. For instance, integrating EEG data with structural MRI (sMRI) could improve precision. This hybrid method may offer practical advantages while maintaining clinical feasibility.

As neurological patterns in patients with neurological disorders can evolve over time [67]– [68], the development of personalized models that dynamically adapt to these individual changes is important, as implemented in this study. However, in clinical practice, accurately identifying motor hotspots using TMS-based MEPs is not always feasible, particularly in patients with impaired corticospinal excitability. In such cases, a generalized model may provide a practical alternative by enabling the identification of stimulation targets without relying on MEP-based ground truth. To this end, we will focus on enhancing model generalizability by applying domain adaptation techniques and exploring hybrid modeling strategies that integrate the strengths of both personalized and generalized approaches.

Although previous studies have reported that stimulating individual motor hotspots is more effective than targeting C3 and C4 [26, 65], further clinical validation is needed to confirm whether applying tES to motor hotspots identified by our EEG-based approaches is effective. Especially, given that EEG patterns in stroke patients are influenced by the severity and location of the lesion, further investigation should be conducted with varied sample cohorts, considering variables such as the severity of the stroke (e.g., severe, moderate, mild) and the duration of onset (e.g., acute, chronic), to ensure that our findings are generalizable across different patient profiles. Finally, although we employed an appropriate study design and statistical analysis methods to draw meaningful conclusions, the limited size of our dataset may restrict the generalizability of our findings. **Based on a post hoc power analysis**,** assuming a medium effect size (Cohen’s d = 0.5)**,** a significance level of 0.05**,** and a two-tailed test**,** the statistical power with a group size of 30 was estimated to be approximately 47.8%**,** suggesting that the current sample size may be underpowered. To achieve the conventional target power of 80%**,** approximately 64 subjects per group would be required. Although statistically significant results were obtained**,** this limitation should be considered when interpreting the findings. Future studies should incorporate a priori power analyses to ensure sufficient sample sizes during the planning phase.** To address this limitation, we plan to validate our findings through future studies involving larger and more diverse populations.

Beyond motor rehabilitation, tES extends beyond exercise rehabilitation and can be employed for enhancing cognitive functions and mitigating symptoms of mental illness [69–73]. Typically, for cognitive rehabilitation, tES electrodes are attached on F3 and F4 to stimulate the dorsolateral prefrontal cortex (DLPFC), which plays a crucial role in various cognitive functions [74]– [75]. However, similar to the individual variability observed in the optimal motor hotspot locations, there is likely also individual variability in the target locations for tES in cognitive rehabilitation. Therefore, we suggest that the proposed algorithm could be adapted for a broader range of applications, including cognitive rehabilitation and alleviation of mental illness symptoms.

## Conclusion

In this study, we introduced an advanced EEG-based motor hotspot identification using CNN and confirmed the practicality of our proposed algorithm by examining its performance with reduced numbers of channels and trials. Furthermore, we demonstrated the potential clinical application of our algorithm by utilizing EEG data from stroke patients, with a mean error distance of less than 2 mm.

## Supplementary Information

Supplementary Material 1

## Data Availability

The datasets are available from the corresponding authors on reasonable request.

## Abbreviations

TMS: Transcranial magnetic stimulation
tES: Transcranial electrical stimulation
EEG: Electroencephalography
CNN: Convolutional neural network
WHO: World Health Organization
tDCS: Transcranial direct current stimulation
tACS: Transcranial alternating current stimulation
MEP: Maximum motor evoked potential
EMG: Electromyography
IRB: Institutional Review Board
FDI: First dorsal interosseous
CAR: Common average re-reference
ICA: Independent component analysis
PSD: Power spectral density
ECoG: Electrocorticography
FFT: Fast Fourier transform
ReLU: Rectified linear unit
ADAM: Adaptive moment
MSE: Mean square error
K-MMSE: Korean Mini-Mental State Exam
ANN: Artificial neural network
DLPFC: Dorsolateral prefrontal cortex
