# Supplementary material for: Clinical feasibility of motor hotspot localization based on electroencephalography using convolutional neural networks in stroke
Source: J Neuroeng Rehabil. 2025 Sep 26;22:193. doi: 10.1186/s12984-025-01736-3 (PMC12465479; doi:10.1186/s12984-025-01736-3)
Supplement: Supplementary file 1 — Supplementary Material 1 [file 12984_2025_1736_MOESM1_ESM.docx]

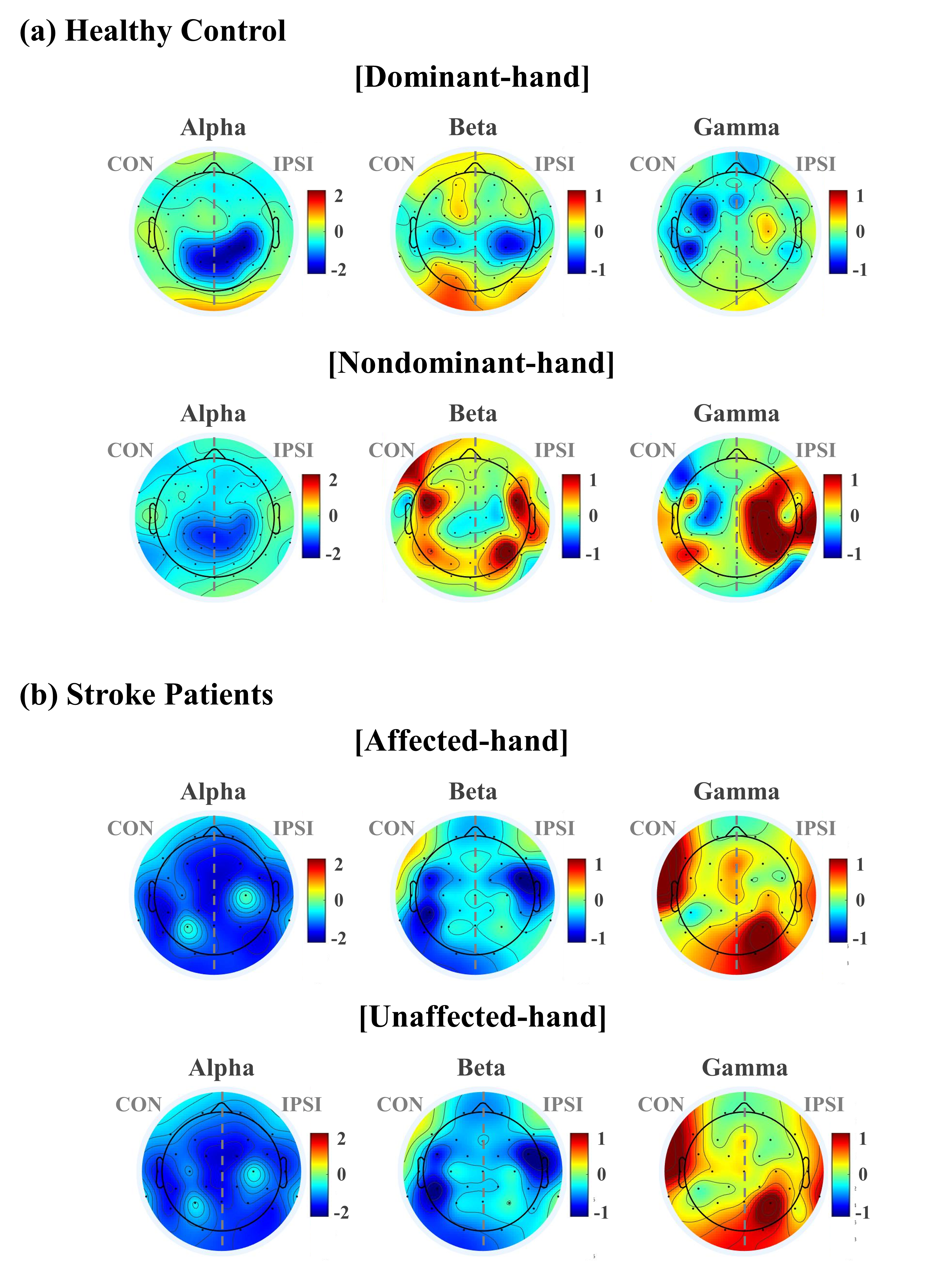


Figure 1. Grand averaged topographical event-related spectral perturbation (ERSP) maps for alpha (8-13 Hz), beta (13-30 Hz), and gamma (30-50 Hz) bands associated with voluntary movements in (a) healthy group and (b) stroke patient group. Despite the strong ERD pattern observed around the motor area in the alpha and beta bands of healthy group, the stroke group shows a comparatively widespread pattern regardless of the hemiplegia site. Furthermore, the gamma band activity in the motor area shows clear laterality in the healthy group, which is not observed in the stroke group. The terms CON and IPSI refer to the contralateral and the ipsilateral hemispheres, respectively, with respect to the hand being used.

Table 1. Summary of performance according to hyperparameters using raw data from healthy individuals. The options highlighted in red indicate the final selections. For more detailed information on the finalized options, please refer to section 2.4.2 in the Methods and Table 2 in the main article. Note that a term of NaN indicates that the experimenter has empirically halted the training process, as determined by monitoring the loss value.

| **Parameters** | | **Error distance (mm)** |
| --- | --- | --- |
| **Kernel size (k)** | **10** | **8.20 ± 21.80** |
|  | **7** | **2.20 ± 0.50** |
|  | **5** | **5.60 ± 14.40** |
|  | **3** (initial) | **2.70 ± 2.30** |
| **Optimizer** | **Adam** (initial) | **2.70 ± 2.30** |
|  | **SGD** | **NaN** |
| **Activation** | **ReLU** (initial) | **2.70 ± 2.30** |
|  | **ELU** | **32.19 ± 62.35** |
| **Learning rate** | **0.1** | **NaN** |
|  | **0.01** (initial) | **2.70 ± 2.30** |
|  | **0.001** | **1.02 ± 0.51** |
| **Batch normalization** | **Yes** | **131.94 ± 201.06** |
|  | **No** (initial) | **2.70 ± 2.30** |


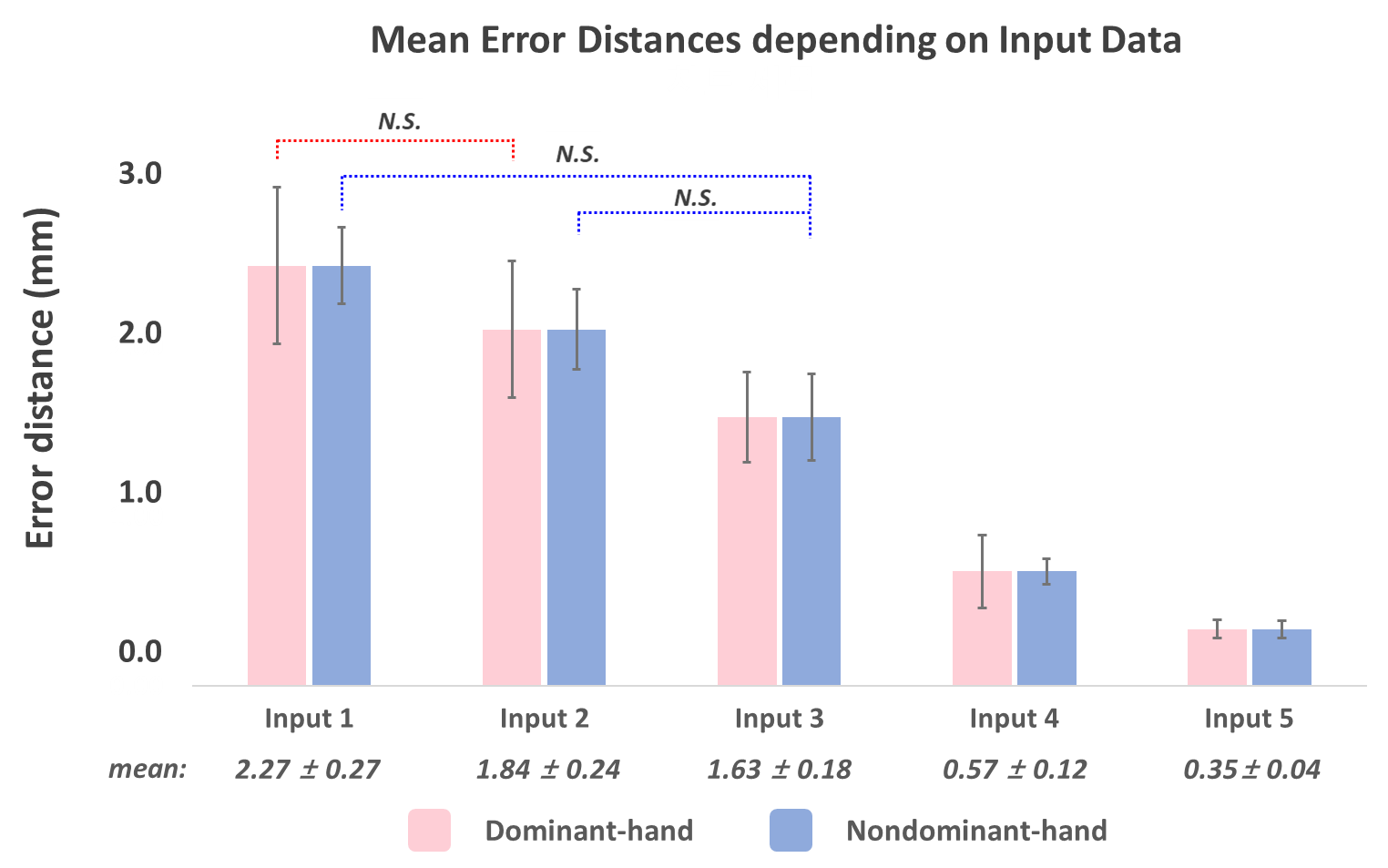


Figure 2. Mean error distances and standard errors in identifying motor hotspot locations with respect to input data (Friedman test with Bonferroni corrected, *p*-value < 0.05: N.S. indicates no significance. All other pairs show significant differences.). Note that no significant difference was observed between the dominant and nondominant hands for all conditions (Wilcoxon rank sum, p-value < 0.05).


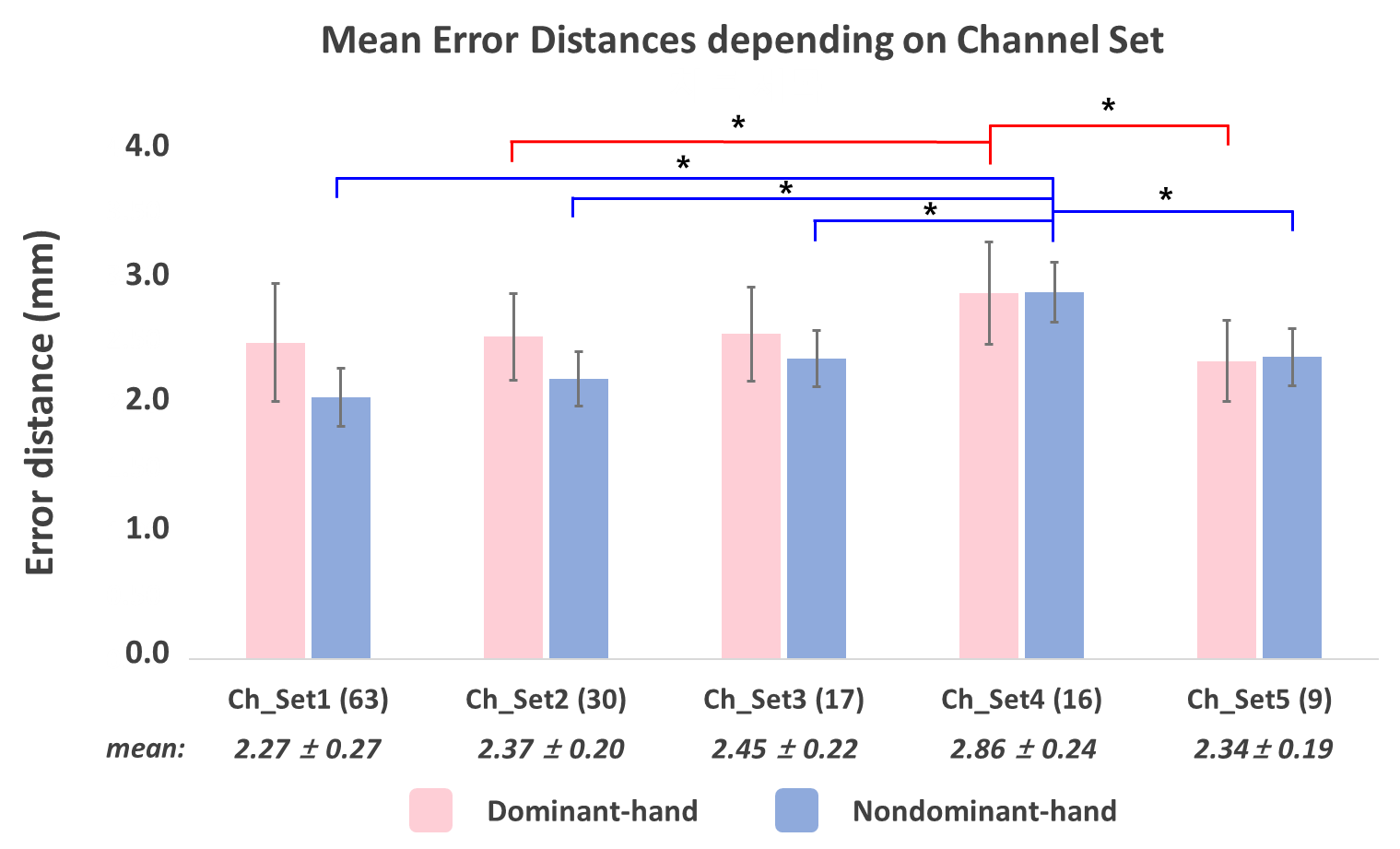
 Figure 3. Mean error distances and standard errors in identifying motor hotspot locations using raw-EEG with respect to the number of channels (Friedman test with Bonferroni corrected, *p*-value < 0.05). Note that no significant difference was observed between the dominant and nondominant hands for all conditions (Wilcoxon rank sum, p-value < 0.05).


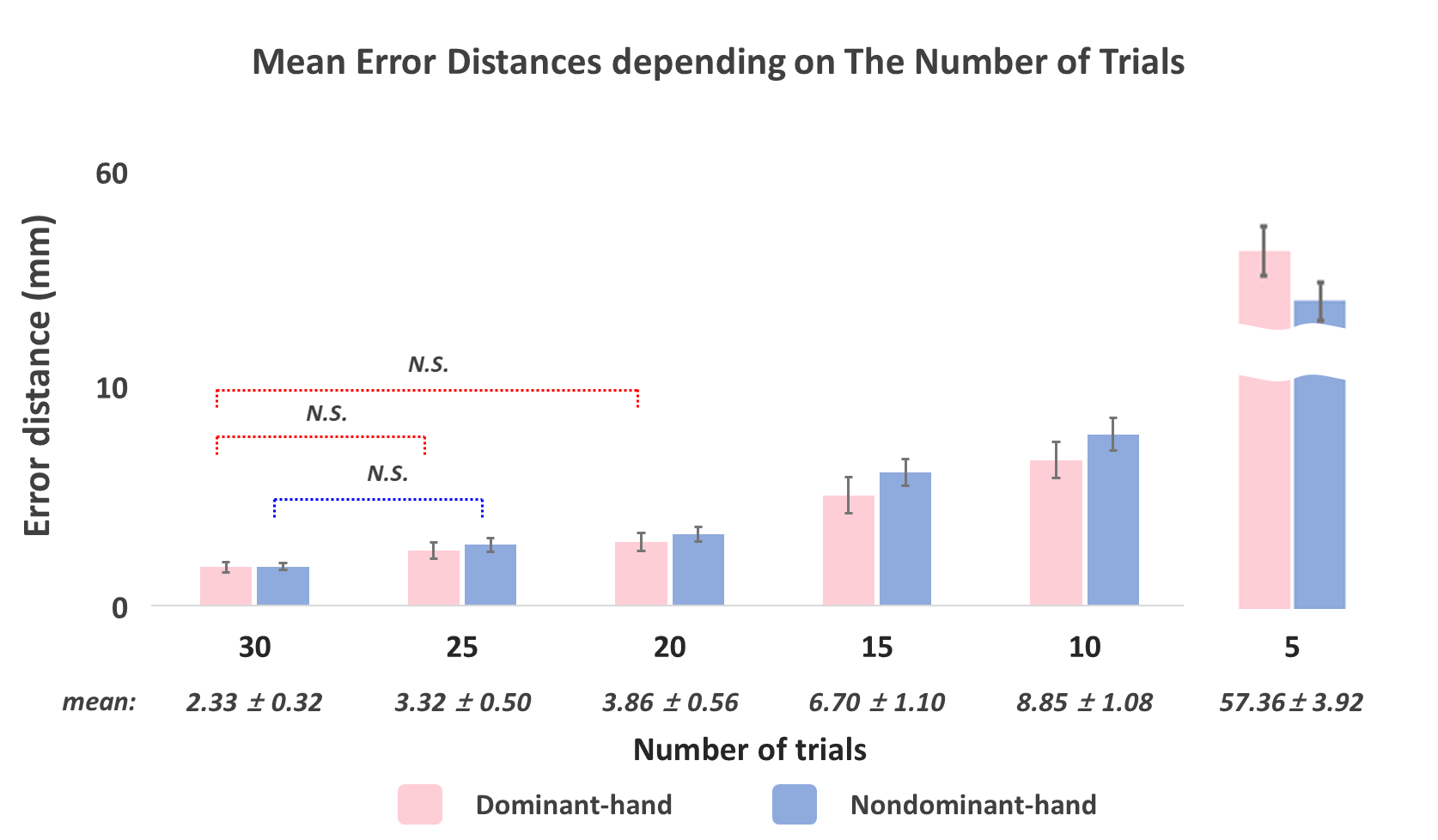


Figure 4. Mean error distances and standard errors in identifying motor hotspot locations using 9 channels of raw EEG with respected to the number of trials (Friedman test with Bonferroni corrected, p-value < 0.05: N.S. indicates no significance. All other pairs show significant differences.). Note that no significant difference was observed between the dominant and nondominant hands for all conditions (Wilcoxon rank sum, p-value < 0.05).


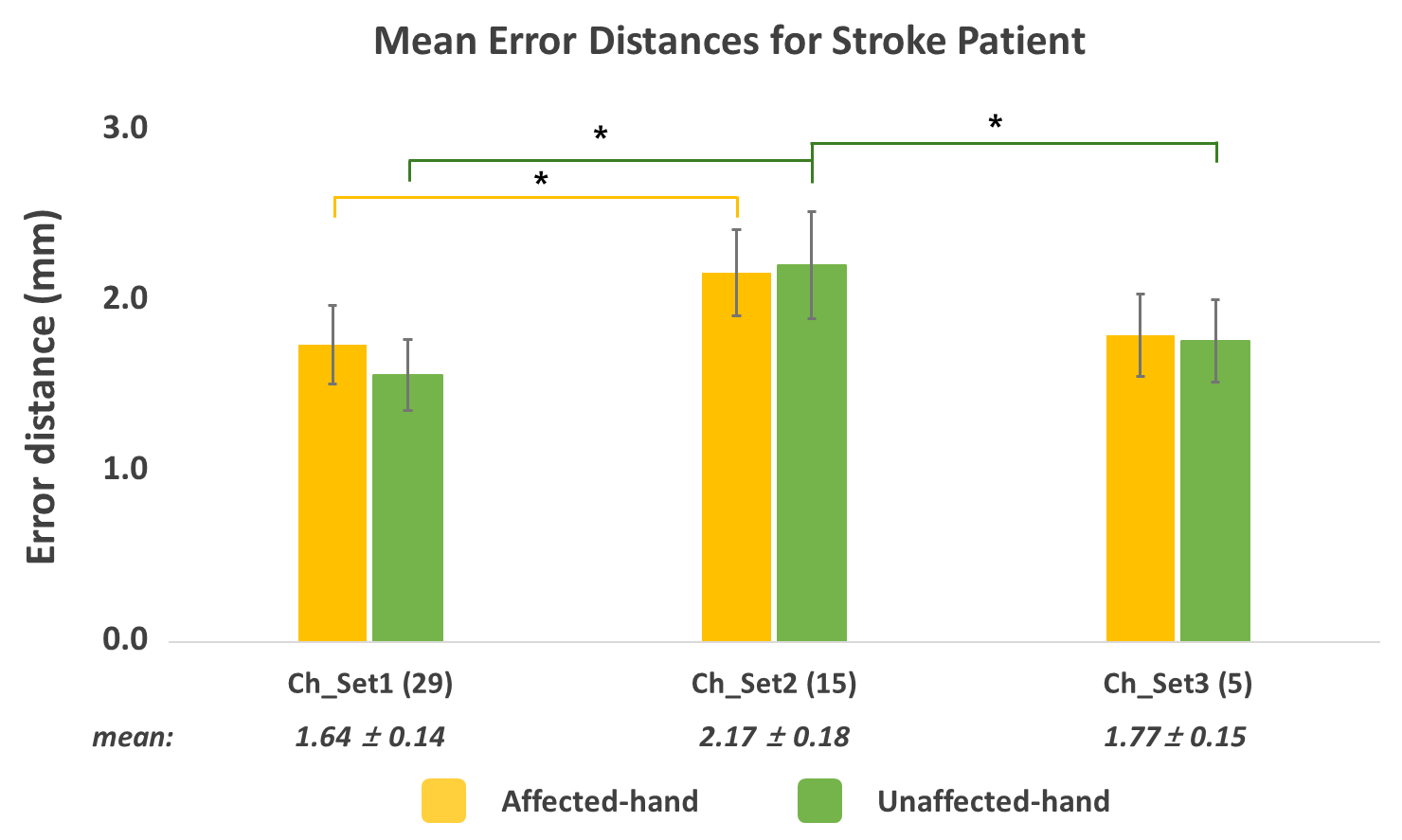


Figure 5. Mean error distances and standard errors in identifying motor hotspot locations using raw EEG from the stroke patient with respect to the number of channels (Friedman test with Bonferroni corrected, p-value > 0.05). Note that no significant difference was observed between the affected and unaffected hands for all conditions (Wilcoxon rank sum, p-value < 0.05).


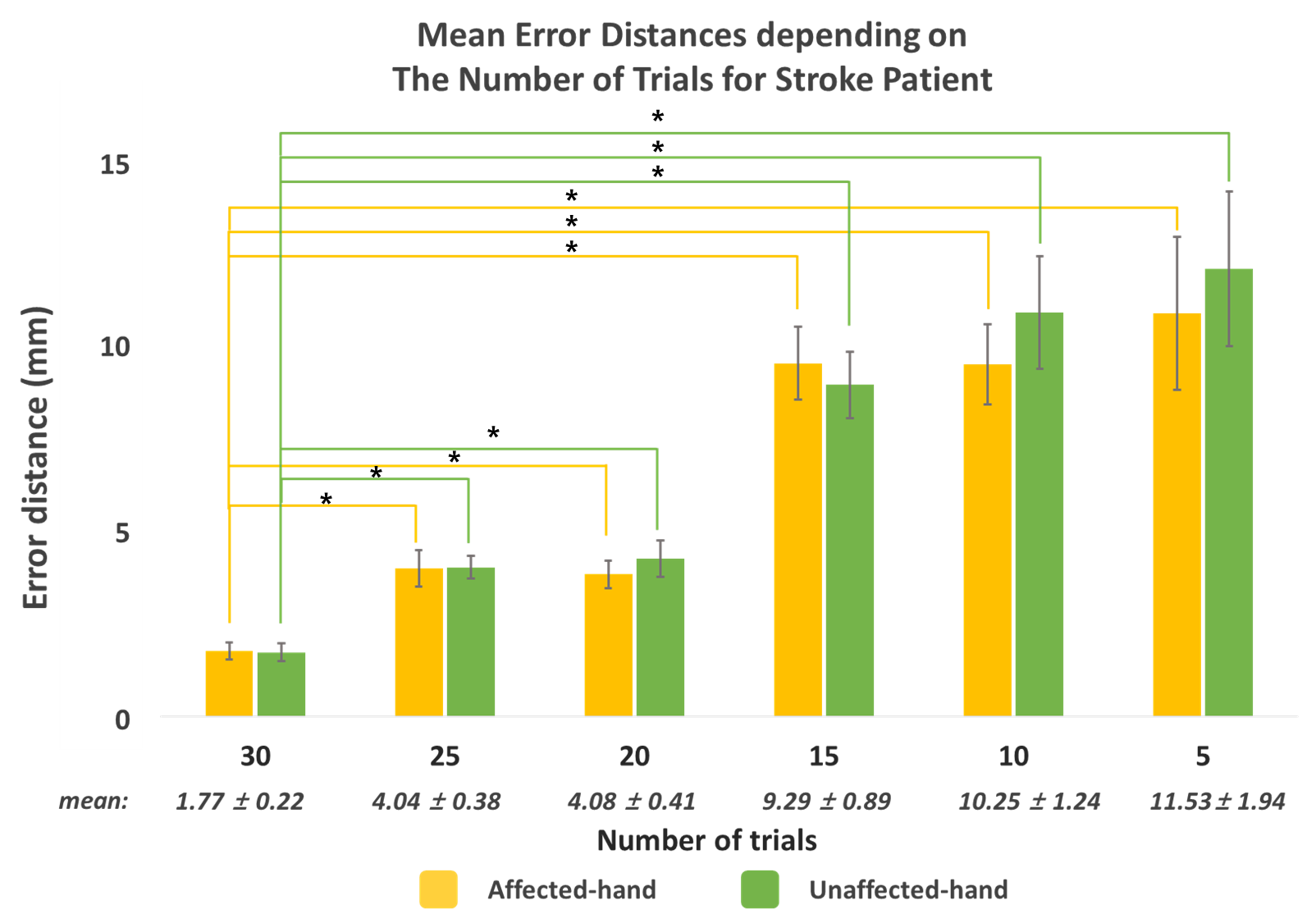


Figure 6. Mean error distances and standard errors in identifying motor hotspot locations using 5 channels of raw EEG from the stroke patient with respected to the number of trials (Friedman test with Bonferroni corrected, p-value < 0.05: N.S. means no significance, and the pairs without N.S. means significant differences). Note that no significant difference was observed between the affected and unaffected hands for all conditions (Wilcoxon rank sum, p-value < 0.05).
